# Weight Status of American Indian and White Elementary School Students Living in the Same Rural Environment, Oklahoma, 2005-2009

**DOI:** 10.5888/pcd9.110233

**Published:** 2012-03-29

**Authors:** Amanda E. Janitz, William E. Moore, Aietah L. Stephens, Kathryn E. Abbott, June E. Eichner

**Affiliations:** University of Oklahoma Health Sciences Center, Oklahoma City, Oklahoma; University of Oklahoma Health Sciences Center, Oklahoma City, Oklahoma; University of Oklahoma Health Sciences Center, Oklahoma City, Oklahoma; Anadarko Elementary Schools, Anadarko, Oklahoma; University of Oklahoma Health Sciences Center

## Abstract

**Introduction:**

Studies have assessed rates of childhood obesity in diverse populations, but few have been able to compare the weight status of American Indian and white children living in the same community and attending the same schools. The objective of this study was to measure and compare the weight status of American Indian and white elementary school students (kindergarten through 5th grade) from 2005 through 2009 in an Oklahoma school district.

**Methods:**

We assessed height, weight, age, and sex to calculate body mass index, body mass percentile, and categorical weight status of students, based on the Centers for Disease Control and Prevention 2000 Growth Charts. We used binomial regression to generate risk ratios (RRs) to compare student weight status by race, sex, and age.

**Results:**

An average of 753 students was measured in each year; mean age was 8.3 years. From 2005 through 2009, 45.4% of American Indian students and 65.1% of white students were healthy weight or underweight. Greater proportions of American Indian children were very obese (weighted average RR, 2.0); obese (weighted average RR, 1.6), or overweight (weighted average RR, 1.8) compared with white children. The overall prevalence of excess weight changed little during the study period.

**Conclusion:**

American Indian children had a greater risk of being overweight, obese, or very obese than white children from the same rural environment.

## Introduction

American Indian adults are not generally sampled in large enough numbers for valid statistical inference and comparison in national studies because of their small numbers in the population. American Indian children are also not adequately sampled in national studies. Many American Indians live in Oklahoma communities with other racial groups, allowing for comparisons that hold the geographic macroenvironment constant while possibly minimizing, though not eliminating, other potential confounders, such as socioeconomic status and regional cultural differences.

Many American children live in an obesogenic environment, regardless of whether they live in subsidized urban housing, suburban residences, or small farms in rural communities. The availability of fast food, large portion sizes, energy-dense food, labor-saving devices, screen time, and automobiles and the increased frequency of eating make survival convenient and sedentary and obesity endemic (1-3). Although the environmental risk factors for obesity are becoming endemic worldwide, disparities in risk associated with culture, geography, and affluence can also affect risk. Some of the risk factors of the built environment, such as a high density of fast-food restaurants and a lack of facilities or pleasant places to exercise, have been shown to be positively associated with the risk for obesity (4,5). While many schools, communities, and health departments are trying to alter this situation, the prevailing influence is to move less and eat more.

Reports from the National Health and Nutrition Examination Survey (NHANES) have indicated a possible leveling of the prevalence of overweight or obesity among children. However, evidence also suggests that the proportion of children who are very obese (≥97th percentile of the Centers for Disease Control and Prevention [CDC] 2000 Growth Charts) is increasing, particularly among African American and Hispanic children (6,7). American Indian children, like their racial/ethnic minority and nonminority counterparts, are also at risk (8-10). Environmental pressures, poverty and unemployment, and susceptibility to metabolic disease do not bode well for their future health (11). Rates of type 2 diabetes among American Indian adults are already among the highest for population groups in the United States (12,13).

The objective of this study was to assess and compare the weight status of a sample of American Indian and white students residing in the same rural environment and attending 3 elementary schools in Anadarko, Oklahoma, to ascertain differences by race, sex, or age.

## Methods

### Study design

We measured the height and weight of children in kindergarten through 5th grade in 3 elementary schools in Anadarko, Oklahoma, annually for 5 consecutive years, 2005 through 2009. We calculated body mass index (BMI) and categorized weight status by race, sex, and age. This study was approved by the institutional review board of the University of Oklahoma Health Sciences Center.

### Study setting

Anadarko is a small, multiracial/ethnic town of 6,762 people in southwestern Oklahoma (14). Seven tribes, Apache, Caddo, Comanche, Delaware, Fort Sill Apache, Kiowa, and Wichita, have tribal headquarters in the region. According to the 2010 US Census, 48.6% of the population of Anadarko are American Indian/Alaska Native, 33.1% are white, and 5.5% are a combination of American Indian/Alaska Native and white (15). The Anadarko school district has 3 public elementary schools; each school has only 2 grades. One school is for kindergarten and 1st grade, one is for 2nd and 3rd grade, and one is for 4th and 5th grade. The students in the elementary schools are predominantly American Indian (68.5%); 20.0% are non-Hispanic white, 6.6% are white Hispanic, 4.4% are African American, and 0.5% are Asian/Pacific Islander. From 2005 through 2009, 79.4% of American Indian students and 47.5% of white students were eligible for free or reduced-price lunch.

### Participant recruitment

All ambulatory children enrolled in the 3 schools were eligible to participate in this study. The measurement of height and weight of all students was included as part of the school's general health screening. When students enrolled for the school year, each parent/guardian received a letter stating that health screenings performed that year would include the measurement of height and weight and hearing, vision, and other screenings. Parents had the option to decline these measurements and screenings. Children who did not have signed parental permission were not measured or screened.

### Measures

We calculated BMI as weight (kg) divided by height (m^2^) by using CDC's 2000 Growth Charts for the United States. We measured weight rounded to the nearest 0.1 kg by using a Tanita 800 digital scale (Tanita Corporation of America, Inc., Arlington Heights, Illinois). We measured height rounded to the nearest 0.1 cm by using a Perspective Enterprises stadiometer (Perspective Enterprises, Portage, Michigan). Children removed their shoes and outerwear for measurement. We obtained data on age and race/ethnicity from the school administration.

### Statistical analysis

We excluded African American, Asian/Pacific Islanders, and Hispanic students because of their small numbers. We performed statistical analyses only on American Indian and non-Hispanic white children, the 2 most numerous racial groups. Although we did not anticipate any differences (16), we performed goodness-of-fit analyses to determine whether participating students differed from the overall elementary school population in the distribution of race or sex. We calculated response rates for each year by dividing the number of children who participated in the study by the number of children who enrolled at the beginning of the school year.

We categorized the weight status of each student using CDC 2000 Growth Charts: underweight (<5th percentile); healthy weight (5th to <85th percentile); overweight (85th to <95th percentile); obese (95th to <97th percentile); and very obese (≥97th percentile). We used binomial regression with risk ratios (RRs) to evaluate differences in weight status by race, sex, and age within each school year. We categorized weight status in 3 ways for the binomial regression: very obese versus healthy weight or underweight as the referent; obese versus healthy weight or underweight as the referent; and overweight versus healthy weight or underweight as the referent.

Data with individual identifiers for longitudinal analysis were not available. Even if such data were available, the amount of data would be greatly reduced. In addition to normal attrition caused, for example, by families moving out of the community, approximately 17% of each cohort ages out of elementary school each year. Because we know many children were measured in multiple years, the assumption of independence is not valid, and we are precluded from inferential analysis. However, we did calculate weighted averages of RRs for the study period to summarize the results. We used a *P* value of .05 for all inferential analyses to denote statistical significance.

We used Epi Info version 3.5.1 (CDC, Atlanta, Georgia), to generate BMI, BMI percentile, and BMI *z* scores. We analyzed the data using Stata software version 11 (StataCorp, College Station, Texas).

## Results

We measured a similar number (average, 753; range, 706 to 795) of students each year (Table 1). Response rates averaged 93%. The mean age at measurement was 8.3 years (range, 5.1-12.4 y). We found no sex or race differences between children who participated in the study and the overall school population. Between 75% and 80% of participants were American Indian, and 21% to 25% were white. Approximately half of the participants were girls. Averaged over 5 years, 45.4% of American Indian students and 65.1% of white students were healthy weight or underweight. Few children (either American Indian or white) were underweight. American Indian students had a higher prevalence of being overweight, obese, or very obese than white students for each year of analysis (Table 1 and Table 2).

The percentage of American Indian children each year who were very obese (range, 25.5% to 28.2%) was greater than the percentage who were overweight (range, 17.6% to 22.3%) or obese (range, 6.1% to 8.7%). During the 5-year study period, 24.2% (weighted average) of all students were very obese (26.9% American Indian, 14.7% white).

When we controlled for sex and age and compared very obese children with children who had a healthy weight or were underweight, American Indian children had a significantly higher risk (RR weighted average, 2.0) for being very obese than white participants in each year (Table 3). When we controlled for race and age, sex was not significantly associated with risk for being very obese in any year. Increasing age was significantly associated with the risk for being very obese in 4 of 5 years.

When we controlled for sex and age and compared obese children with children who had a healthy weight or were underweight, American Indian children had a higher risk (RR weighted average, 1.6) for being obese than white children in each year. However, this risk was significantly higher only in 2005 (Table 3). When we controlled for race and age, sex was not significantly associated with risk for being very obese in any year. Increasing age was significantly associated with the risk for being obese only in 2008.

When we controlled for sex and age and compared overweight students with children who had a healthy weight or were underweight, American Indian children had a significantly higher risk (RR weighted average, 1.8) for being overweight than white students in each year (Table 3). When we controlled for race and age, boys had a significantly higher risk for being overweight than girls only in 2006. Age was not significantly associated with the risk for being overweight in any year.

## Discussion

Averaged over 5 years and for each year studied, American Indian elementary school students within 1 school district in a rural Oklahoma community had an increased risk of being overweight, obese, or very obese compared with their white peers; 24.2% (weighted average) of all students were very obese (26.9% American Indian, 14.7% white).

In 1 year, boys had a higher risk of being overweight than girls, and older age was sometimes significantly associated with an increased risk of being overweight or heavier. Higher percentages of American Indian children were very obese than were obese or overweight. Although these analyses were cross-sectional, they show that obesity has become an endemic condition for children in this community, and American Indian children are at greatest risk.

The increased prevalence of obesity among children and its effects in both childhood and adulthood have been documented (17-21). One study reported that children who have high levels of blood glucose were more likely to have a high BMI *z* score in childhood and adulthood and continue to have elevated levels of blood glucose (22). Another study found that BMI *z* score was significantly related to indicators of poor health, including higher levels of cholesterol, insulin, blood pressure, and liver enzymes (23). Among children in the Anadarko school district, 1 study showed that higher BMI is associated with higher risk for elevated blood pressure (24). Children in the highest quartile of BMI have also been shown to have an increased risk for premature death (25).

Using NHANES data from 2007-2008, Ogden et al reported the weight status of children aged 6 to 11 (6). Using their categories (BMI ≥85th percentile, BMI ≥95th percentile, and BMI ≥97th percentile), we compared their data with ours for 2007. The percentage of children in our study population was higher than the percentage in the NHANES population for the overweight or greater BMI category. In our study population, 51.7% of the children had a BMI equal to or greater than the 85th percentile, compared with 35.5% of the NHANES children; 32.0% of our children had a BMI equal to or greater than the 95th percentile, compared with 19.6% of the NHANES children; and 23.7% of our children had a BMI equal to or greater than the 97th percentile, compared with 14.5% of the NHANES children. These data show that the prevalence of obesity among our study population is greater than the national prevalence, and it is skewed toward the most extreme category of obesity.

Our study had several limitations. First, our data are cross-sectional. The data provide a snapshot of the weight status of children at 3 elementary schools for each of 5 consecutive years and therefore, do not indicate changes for each child over time. Second, we did not collect data for each student on other possible risk factors for obesity, such as poverty (eg, eligibility for free or reduced-price school lunch). But the aggregate difference in eligibility among our study population (79.4% of American Indian students and 47.5% of white students) as a surrogate for poverty may account for some of the obesity risk among the American Indian students. Data on other potential risk factors or confounders for obesity, such as neighborhood and proximity to exercise venues, were not available.

The strengths of our study were a high response rate, the collection of data over 5 years, and the ability to compare — by race — children living and attending schools in the same school district (ie, the same rural environment). These 3 factors helped eliminate the possibility that regional culture or school environment played a role in creating disparities in obesity among the study population. Our study demonstrated a consistently high percentage of children who were overweight, obese, or very obese, and American Indian children were shown to be at greater risk for obesity than white children.

Additional studies into the greater risk among American Indian children are warranted, and such studies should collect data on variables that can rule out socioeconomic and other environmental causes of obesity. This study makes clear the urgent need for interventions to improve nutrition, increase physical activity, and minimize sedentary behavior in schools and communities.

## Figures and Tables

**Table 1.** Characteristics of Children in Study on Body Mass Index of American Indian and White Elementary School Students in Oklahoma, 2005-2009

Characteristic	2005	2006	2007	2008	2009
**Response rate^a^, %**	97.7	97.5	86.6	96.6	91.3
**No. of children measured**					
Total	795	755	706	731	780
White girls	95	87	78	80	77
White boys	104	94	85	73	80
American Indian girls	298	277	271	281	292
American Indian boys	298	297	272	297	331
**Children overweight or heavier^b^, %**					
Total	47.6	50.5	51.7	52.1	49.4
White girls	31.6	32.2	29.5	35.0	28.6
White boys	33.7	44.7	38.8	35.6	38.8
American Indian girls	50.0	50.5	56.1	53.0	51.0
American Indian boys	55.0	57.6	57.7	59.9	55.3

a Number of children measured divided by number of children enrolled at the beginning of the school year.

b Overweight, obese, or very obese (≥85th percentile of Centers for Disease Control and Prevention's 2000 Growth Charts).

**Table 2.** Percentage of Children in Each Body Mass Index Category^a^, by Race, in Study of American Indian and White Elementary School Students in Oklahoma, 2005-2009

Weight Status	2005		2006		2007		2008		2009	

	American Indian	White	American Indian	White	American Indian	White	American Indian	White	American Indian	White
Underweight	0.2	3.0	0.2	1.7	0.6	1.8	0.2	2.0	0.2	0.6
Healthy weight	47.3	64.3	45.6	59.7	42.5	63.8	43.3	62.8	46.6	65.6
Healthy weight or underweight	47.5	67.3	45.8	61.4	43.1	65.6	43.5	64.8	46.8	66.2
Overweight	17.6	14.1	18.8	13.8	21.4	14.1	22.3	13.7	21.0	14.0
Obese	7.7	4.0	8.4	6.1	8.7	7.4	6.1	7.2	6.7	7.6
Very obese	27.2	14.6	27.0	18.8	26.9	12.9	28.2	14.4	25.5	12.1
Overweight, obese, or very obese	52.5	32.7	54.2	38.7	57.0	34.4	56.6	35.3	53.2	33.7

a Determined by Centers for Disease Control and Prevention's 2000 Growth Charts: underweight, <5th BMI percentile; healthy weight, ≥5th percentile and <85th percentile; overweight, ≥85th percentile and <95th percentile; obese, ≥95th percentile and <97th percentile; very obese, ≥97th percentile.

**Table 3.** Binomial Regression Risk Ratios (95% Confidence Intervals) for Body Mass Index Categories^a^ With Independent Variables of Race, Sex, and Age in Study of American Indian and White Elementary School Students in Oklahoma, 2005-2009

Very Obese Compared With Healthy Weight or Underweight					
**Independent variable**	2005 (n = 608)	2006 (n = 563)	2007 (n = 508)	2008 (n = 535)	2009 (n = 573)

**Race**					
American Indian	2.05 (1.45-2.91)	1.59 (1.16-2.18)	2.34 (1.56-3.52)	2.16 (1.46-3.21)	2.31 (1.50-3.56)
White	1 [Referent]	1 [Referent]	1 [Referent]	1 [Referent]	1 [Referent]
**Sex**					
Boys	1.11 (0.88-1.39)	1.18 (0.93-1.48)	1.26 (0.98-1.6)	1.14 (0.91-1.43)	1.14 (0.90-1.45)
Girls	1 [Referent]	1 [Referent]	1 [Referent]	1 [Referent]	1 [Referent]
**Age**	1.12 (1.05-1.19)	1.07 (1.01-1.14)	1.05 (0.98-1.12)	1.08 (1.02-1.15)	1.11 (1.04-1.19)

**Obese Compared With Healthy Weight or Underweight**					

**Independent variable**	**2005 (n = 471)**	**2006 (n = 433)**	**2007 (n = 400)**	**2008 (n = 396)**	**2009 (n = 396)**

**Race**					
American Indian	2.52 (1.22-5.19)	1.73 (0.93-3.21)	1.73 (0.95-3.13)	1.25 (0.66-2.36)	1.25 (0.68-2.29)
White	1 [Referent]	1 [Referent]	1 [Referent]	1 [Referent]	1 [Referent]
**Sex**					
Boys	1.12 (0.68-1.85)	1.04 (0.65-1.67)	0.86 (0.54-1.38)	1.23 (0.72-2.13)	1.22 (0.74-2.01)
Girls	1 [Referent]	1 [Referent]	1 [Referent]	1 [Referent]	1 [Referent]
**Age**	1.13 (0.99-1.29)	1.04 (0.91-1.19)	1.13 (0.99-1.28)	1.19 (1.03-1.37)	1.12 (0.98-1.29)

**Overweight Compared With Healthy Weight or Underweight**					

**Independent variable**	**2005 (n = 550)**	**2006 (n = 507)**	**2007 (n = 480)**	**2008 (n = 500)**	**2009 (n = 548)**

**Race**					
American Indian	1.58 (1.09-2.30)	1.57 (1.07-2.31)	1.85 (1.24-2.77)	1.93 (1.28-2.92)	1.79 (1.19-2.68)
White	1 [Referent]	1 [Referent]	1 [Referent]	1 [Referent]	1 [Referent]
**Sex**					
Boys	1.15 (0.86-1.54)	1.47 (1.09-1.99)	0.95 (0.72-1.25)	1.18 (0.90-1.53)	1.16 (0.89-1.52)
Girls	1 [Referent]	1 [Referent]	1 [Referent]	1 [Referent]	1 [Referent]
**Age**	1.08 (0.99-1.16)	1.0 (0.95-1.1)	0.97 (0.90-1.0)	0.99 (0.92-1.07)	1.05 (0.98-1.13)

a Determined by Centers for Disease Control and Prevention's 2000 Growth Charts: underweight, <5th BMI percentile; healthy weight, ≥5th percentile and <85th percentile; overweight, ≥85th percentile and <95th percentile; obese, ≥95th percentile and <97th percentile; very obese, ≥97th percentile.
